# Clinical outcomes before and after the introduction of clazosentan in aneurysmal subarachnoid hemorrhage: a multicenter retrospective study of clipping and endovascular treatment

**DOI:** 10.1007/s10143-026-04469-6

**Published:** 2026-09-04

**Authors:** Issei Takeuchi, Shinsuke Muraoka, Fumie Kinoshita, Takashi Izumi, Kazuki Ishii, Masahiro Nishihori, Shunsaku Goto, Ryuta Saito

**Affiliations:** 1 Department of Neurosurgery, Nagoya University Graduate School of Medicine, Nagoya, Aichi 466-8560 Japan; 2 Department of Advanced Medicine, Nagoya University Hospital, Nagoya, Aichi Japan; 3 Department of Neurosurgery, Nagoya Ekisaikai Hospital, Nagoya, Aichi Japan

**Keywords:** Aneurysmal subarachnoid hemorrhage, Endovascular treatment, Clipping, Clazosentan, Fasudil, Cerebral vasospasm

## Abstract

**Objective:**

Definitive treatment for aneurysmal subarachnoid hemorrhage (aSAH) includes microsurgical clipping and endovascular treatment (EVT). In randomized trials such as ISAT and BRAT, EVT showed superiority in short-term outcomes and comparable or favorable long-term results. Delayed cerebral ischemia is one of the most important determinants of clinical outcomes after treatment for aneurysmal subarachnoid hemorrhage caused by ruptured intracranial aneurysms. Although its pathophysiology is multifactorial, cerebral vasospasm has long been considered a major contributing factor. In Japan, fasudil has traditionally been used as a perioperative pharmacological agent for the prevention of cerebral vasospasm; since 2022, clazosentan has also become available. This study aimed to compare outcomes between clipping and EVT before and after the introduction of clazosentan in patients with aSAH.

**Methods:**

Using RECOVER registry data, we analyzed patients with aSAH who underwent definitive treatment with clipping or EVT within 48 h of onset and received either fasudil or clazosentan between 2021 and 2024. The primary endpoint was a favorable functional outcome at discharge, defined as a modified Rankin Scale (mRS) score of 0–2. Secondary endpoints included cerebral vasospasm, delayed cerebral ischemia (DCI), pulmonary complications, hypotension, cerebral edema, and new intracranial hemorrhage. In multivariable analyses, we adjusted for age, World Federation of Neurosurgical Societies (WFNS) grade, Fisher group, anterior circulation aneurysm, and the use of cerebrospinal fluid drainage. The study period was divided into the fasudil era and the clazosentan era, corresponding to the periods before and after the introduction of clazosentan, respectively.

**Results:**

A total of 496 patients were analyzed, of whom 57.5% underwent microsurgical clipping. Overall, favorable outcomes at discharge, defined as an mRS score of 0–2, were significantly more frequent in the EVT group than in the clipping group (59.2% vs 48.4%, *p* = 0.004). In the fasudil era, the EVT group showed a higher proportion of favorable outcomes than the clipping group (59.0% vs 41.5%, *p* = 0.007), whereas no statistically significant between-group difference was observed in the clazosentan era (72.6% vs 62.7%, *p* = 0.15). Among secondary outcomes, the incidence of vasospasm-related DCI was not significantly different between the groups, although it was numerically lower in the EVT group.

**Conclusion:**

Functional outcomes appeared more favorable in both the clipping and EVT groups during the clazosentan era. Across all study periods, the observed proportion of patients with favorable functional outcomes was higher in the EVT group than in the clipping group. However, because formal interaction testing did not identify statistically significant effect modification, the apparent reduction in between-group differences observed in the clazosentan era should be interpreted with caution and considered hypothesis-generating. Further prospective studies are needed to clarify the clinical relevance of these era-based observations.

## Introduction

Aneurysmal subarachnoid hemorrhage (aSAH) is a devastating condition with high morbidity and mortality. Without definitive treatment, 20–30% of patients experience rebleeding within the first month, which markedly worsens prognosis [1]. Microsurgical clipping and endovascular treatment (EVT) are established options for preventing rebleeding, with treatment choice determined based on patient status and aneurysm characteristics [2–8].

Randomized trials, such as the International Subarachnoid Aneurysm Trial (ISAT) and Barrow Ruptured Aneurysm Trial (BRAT), demonstrated better short-term outcomes with EVT, a finding subsequently supported by meta-analyses at 1 year [2–4]. However, long-term follow-up revealed no clear survival advantage for either treatment, while EVT was associated with higher rates of retreatment and rebleeding. [5–8]

Clazosentan, a selective endothelin-A receptor antagonist, was introduced into clinical practice in Japan in 2022. Clinical trials showed that clazosentan reduced vasospasm-related morbidity and mortality compared with placebo [9], and real-world data from our group suggested more favorable outcomes relative to fasudil [10]. These advances have broadened therapeutic strategies, including the use of clazosentan or fasudil alone, combination therapy, and individualized decisions between clipping and EVT based on patient and institutional factors.

Using data from the multicenter retrospective RECOVER registry, we aimed to compare outcomes between patients with aSAH who underwent microsurgical clipping and those who underwent EVT, in the context of perioperative pharmacological management with fasudil or clazosentan. Particular emphasis was placed on differences between the periods before and after the introduction of clazosentan.

## Methods

### Ethical approval and study design

This study was conducted in accordance with the principles of the Declaration of Helsinki and was approved by the Ethics Committee of Nagoya University Hospital (approval number: 2022–0476). This retrospective study used anonymized clinical data, and informed consent was obtained through an opt-out procedure in accordance with institutional ethics approval. Anonymized data and related materials are archived in the Nagoya University repository. This multicenter, retrospective, registry-based observational study compared treatment outcomes of aneurysmal subarachnoid hemorrhage (aSAH) before and after the introduction of clazosentan in patients who underwent microsurgical clipping or endovascular treatment (EVT). All study procedures adhered to the Strengthening the Reporting of Observational Studies in Epidemiology (STROBE) guidelines, and twenty institutions with extensive experience in vasospasm management and active participation in clinical registries contributed to ensure diversity in patient backgrounds while maintaining consistency in treatment practices.

### Study population

Patients with aSAH registered between April 2021 and March 2024 were screened for eligibility. Diagnosis was confirmed according to the American Heart Association/American Stroke Association guidelines using head computed tomography (CT), CT angiography (CTA), and digital subtraction angiography (DSA). Eligible patients were those with a premorbid modified Rankin Scale (mRS) score of 0–2 who were diagnosed with aSAH and underwent definitive treatment—clipping or EVT—within 48 h of onset. To ensure comparability across treatment eras, only patients who received either fasudil or clazosentan following treatment for ruptured aneurysms were included. Patients were excluded if they experienced re-rupture during hospitalization, had incomplete clinical data such as missing discharge mRS or perioperative complication records, or received neither fasudil nor clazosentan.

Treatment eras were defined according to drug availability and use. The fasudil era included patients treated with fasudil before the introduction of clazosentan, whereas the clazosentan era comprised patients treated with clazosentan after its introduction. Accordingly, patients treated with fasudil alone during the clazosentan era were excluded.

### Data collection

Clinical data were collected using a standardized electronic case report form and included demographic characteristics, body mass index, vascular risk factors, premorbid mRS, World Federation of Neurosurgical Societies (WFNS) grade, Fisher group, aneurysm location, treatment modality, use of cerebrospinal fluid drainage, postoperative pharmacological management, cerebral vasospasm, vasospasm-related delayed cerebral ischemia (DCI), brain edema, pulmonary complications, hypotension, de novo intracranial hemorrhage (de novo ICH), and discharge mRS.

### Postoperative management protocol

All patients underwent microsurgical clipping or endovascular treatment (EVT) within 48 h of symptom onset. Clazosentan (continuous intravenous infusion at 10 mg/h) or fasudil (30 mg three times daily) was administered from postoperative day (POD) 1 through POD 14. Other postoperative medications, including cilostazol, statins, and antiepileptic agents (levetiracetam or perampanel), were not uniformly administered to all patients but were initiated at the discretion of the treating physician, typically beginning on POD 1 and continued for 2–3 weeks as needed.

Enteral nutrition or oral intake was generally initiated on POD 1. MRI and MRA were routinely performed on PODs 2 and 10, with additional MRI obtained when new neurological symptoms developed. To evaluate pulmonary complications, daily chest radiographs were obtained during the first postoperative week, and furosemide (20–40 mg/day) was administered when pulmonary edema was identified. Fluid balance was assessed every 24 h until POD 15, and when a negative balance was detected, supplementation with extracellular fluid (500 mL) was provided. In cases of hypotension with preserved urine output, dobutamine (1–5 μg/kg/min) or norepinephrine (0.05–0.3 μg/kg/min) was administered. Clazosentan was discontinued in patients who developed worsening pulmonary complications requiring increased oxygen support or hypotension refractory to vasopressor therapy.

### Imaging assessment

To ensure consistency in the evaluation of cerebral vasospasm, all imaging studies were independently reviewed by two neurosurgeons or radiologists blinded to the clinical course, with disagreements resolved through consensus discussion. Angiographic cerebral vasospasm was defined as a ≥ 25% reduction in vessel diameter compared with the initial preoperative computed tomography angiography (CTA) and was assessed according to each institution’s imaging protocol using magnetic resonance angiography (MRA), CTA, or digital subtraction angiography (DSA). Although statistical measures of interrater agreement, such as Cohen’s κ, were not applied, consistency was maintained through predefined diagnostic criteria and consensus-based interpretation.

DCI was defined as neurological deterioration manifesting as a decrease of ≥ 2 points in the Glasgow Coma Scale (GCS) score that was not attributable to rebleeding, hydrocephalus, electrolyte disturbances, infection, meningitis, seizures, or procedure-related brain injury. Vasospasm-related DCI was defined as a new cerebral infarction or hyperintense lesion on MRI occurring between days 4 and 20 after aneurysmal rupture, absent on initial or immediate postoperative imaging, and considered unlikely to be related to surgical manipulation.

In Japan, postoperative management of aSAH is frequently performed in general wards rather than intensive care units; therefore, routine transcranial Doppler (TCD) monitoring is not universally implemented. Accordingly, DCI surveillance in this study was based on frequent neurological examinations and serial imaging. All patients underwent preoperative CTA and scheduled MRA on postoperative days (PODs) 2 and 10; additional MRI/MRA was obtained when neurological deterioration occurred, and DSA was conducted when cerebral vasospasm was suspected.

### Efficacy and safety assessment

Efficacy was assessed using the mRS score at discharge, with a favorable outcome defined as mRS 0–2.

For safety assessment, the incidence of cerebral vasospasm and vasospasm-related DCI was evaluated using the definitions described above. Vasospasm-related-DCI required vascular narrowing confirmed by CTA or DSA and exclusion of other potential causes, including hypoxic encephalopathy, electrolyte disturbances, infection, meningitis, secondary hydrocephalus, seizure, and procedure-related brain injury [12, 13]. Additional safety endpoints included treatment-related complications such as pulmonary complications, hypotension, brain edema, and de novo intracranial hemorrhage (ICH).

### Statistical analysis

Continuous variables were expressed as medians with interquartile ranges, and categorical variables as frequencies and percentages. Between-group comparisons were performed using a t-test for continuous variables and the chi-square test or Fisher’s exact test for categorical variables. For continuous variables, the median was used as the cutoff value in multivariable analyses.

Multivariable logistic regression analyses were conducted to evaluate efficacy outcomes, adjusting for age, WFNS grade, Fisher grade, use of ventricular drainage, and aneurysm location (anterior circulation). These covariates were selected based on their clinical relevance as established predictors of vasospasm, DCI, and clinical outcome. The same adjustment variables were applied in multivariable analyses of safety outcomes. When logistic regression models failed to converge, Firth’s correction method was applied.

To assess potential effect modification, we additionally performed an interaction analysis by including an interaction term between treatment modality (microsurgical clipping vs. EVT) and drug group (clazosentan vs. fasudil; serving as an era proxy) in the multivariable logistic regression model.

A two-tailed *p*-value < 0.05 was considered statistically significant. Statistical analyses were performed using EZR version 1.64 and SAS version 9.4.

## Results

### Patient characteristics and study population

In total, 583 patients with aSAH who underwent treatment between April 2021 and March 2024 were retrospectively analyzed. Exclusion criteria were as follows: re-rupture during hospitalization (*n* = 9), preexisting disability with an mRS ≥ 3 at onset (*n* = 17), incomplete data (*n* = 45), or absence of treatment with either clazosentan or fasudil (*n* = 16). After these exclusions, 496 patients were included and categorized into the clipping (*n* = 285) and EVT (*n* = 211) groups.

**Fig. 1:**
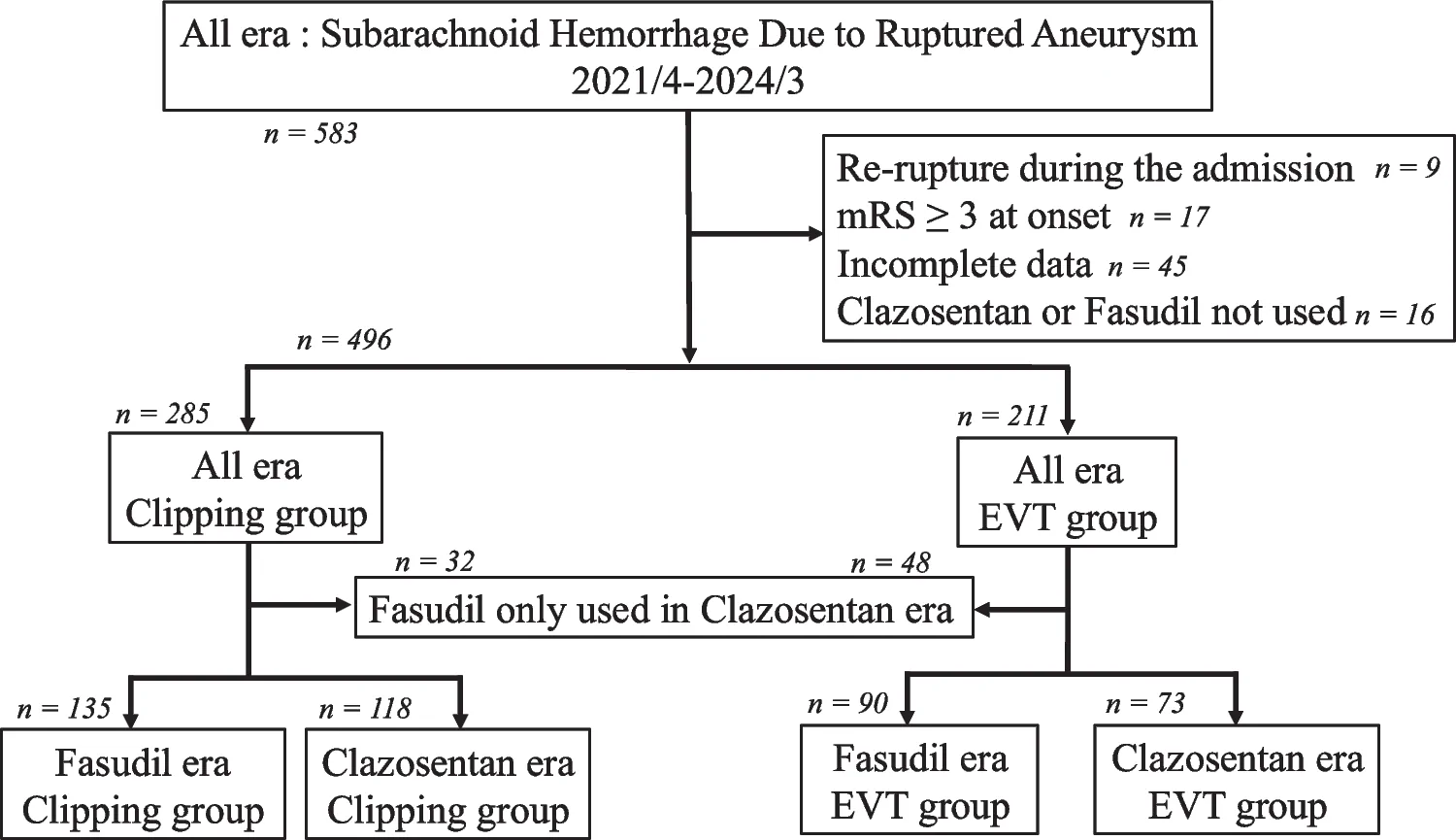
Flowchart of patient selection. Of 583 patients with aneurysmal subarachnoid hemorrhage (aSAH), 87 were excluded owing to re-rupture during admission (*n* = 9), premorbid mRS ≥ 3 (*n* = 17), incomplete data (*n* = 45), or no use of clazosentan/fasudil (*n* = 16). The remaining 496 patients were categorized into the clipping (*n* = 285) and EVT (*n* = 211) groups. Patients treated with fasudil only in the clazosentan era (*n* = 32, 48) were excluded from comparative analyses

To clarify the therapeutic effect of clazosentan, patients treated with fasudil alone during the clazosentan era (EVT: *n* = 48, clipping: *n* = 32) were excluded from the main analysis (Fig. 1). Patients who received clazosentan plus fasudil were included in the analysis, as this was considered a standard treatment pattern during the clazosentan era. Detailed baseline characteristics are summarized in Table 1.

**Table 1:** Patient characteristics

*All Era*				
	All data (*n* = 496)	EVT group (*n* = 211)	Clipping group (*n* = 285)	*p* value
Age, mean (SD)	65 (52–75)	61 (50–75)	67 (53–75)	0.04
Female	330 (66.5%)	138 (65.4%)	192 (67.4%)	0.72
Previous comorbidities				
Hypertension	225 (45.6%)	79 (37.4%)	146 (51.2%)	0.003
Diabetes mellitus	30 (6.1%)	11 (5.2%)	19 (6.7%)	0.63
Dyslipidemia	88 (17.8%)	32 (15.2%)	56 (19.6%)	0.24
aSAH information				
WFNS grade				0.31
Ⅰ	89 (17.9%)	37 (17.5%)	52 (18.2%)	
Ⅱ	148 (29.8%)	66 (31.3%)	82 (28.8%)	
Ⅲ	58 (11.7%)	29 (13.7%)	29 (10.2%)	
Ⅳ	100 (20.2%)	34 (16.1%)	66 (23.2%)	
Ⅴ	101 (20.4%)	45 (21.3%)	56 (19.6%)	
Fisher group				0.01
1	8 (1.6%)	3 (1.4%)	5 (1.8%)	
2	87 (17.5%)	41 (19.4%)	46 (16.1%)	
3	283 (57.6%)	132 (62.6%)	151 (53.0%)	
4	118 (23.8%)	35 (16.6%)	83 (29.1%)	
Aneurysm site (Anterior circulation)	404 (81.5%)	138 (65.4%)	266 (93.3%)	<.001
Treatment information				
CSF drainage	270 (55.0%)	104 (49.3%)	166 (58.2%)	0.09
Used drugs				
Ozagrel	315 (63.5%)	135 (64.0%)	179 (62.8%)	0.86
Cilostazol	321 (64.8%)	136 (64.5%)	184 (64.6%)	1
Statin	266 (54.4%)	117 (55.5%)	149 (52.3%)	0.54

Baseline characteristics were compared between the EVT and clipping groups. Data are presented as median (IQR) or n (%). *P* values were calculated using appropriate tests

*WFNS* world federation of neurosurgical societies, *CSF* cerebrospinal fluid

### Efficacy outcomes

Primary and secondary outcomes for the entire cohort are presented in Table 2. The primary outcome, defined as a favorable functional outcome at discharge (mRS 0–2), was significantly more frequent in the EVT group than in the clipping group (59.2% vs. 48.4%, *p* = 0.004).

**Table 2:** Complications and postoperative outcomes

*All Era*	Univariate				Multivariate	
	EVT group (*n* = 211)	Clipping group (*n* = 285)	*p* value	OR (95% CI)		*p* value
Post mRS score						
mRS score, 0–2	125 (59.2%)	138 (48.4%)	0.02	2.18 (1.28–3.73)		0.004
Complications						
Cerebral vasospasm	41 (19.4%)	91 (31.9%)	0.003	0.65 (0.41–1.05)		0.077
Vasospasm-related DCI	7 (3.3%)	34 (11.9%)	0.001	0.29 (0.12–0.71)		0.006
Brain edema	8 (3.8%)	41 (14.4%)	0.0002	0.26 (0.11–0.63)		0.003
Pulmonary complications	20 (9.5%)	33 (11.6%)	0.55	0.79 (0.4–1.54)		0.49
De novo ICH	1 (0.5%)	14 (4.9%)	0.003	0.14 (0.03–0.65)		0.012
Hypotension	4 (1.9%)	9 (3.2%)	0.57	0.48 (0.11–2.05)		0.32

Data are presented as n (%), as appropriate. Multivariable logistic regression adjusted for age, WFNS grade, Fisher classification, CSF drainage, and aneurysm location (anterior vs posterior circulation). Favorable outcome was defined as mRS 0–2. *DCI* delayed cerebral ischemia, *ICH* intracranial hemorrhage, *mRS* modified rankin scale, *OR* odds ratio, *CI* confidence interval

**Fig. 2:**
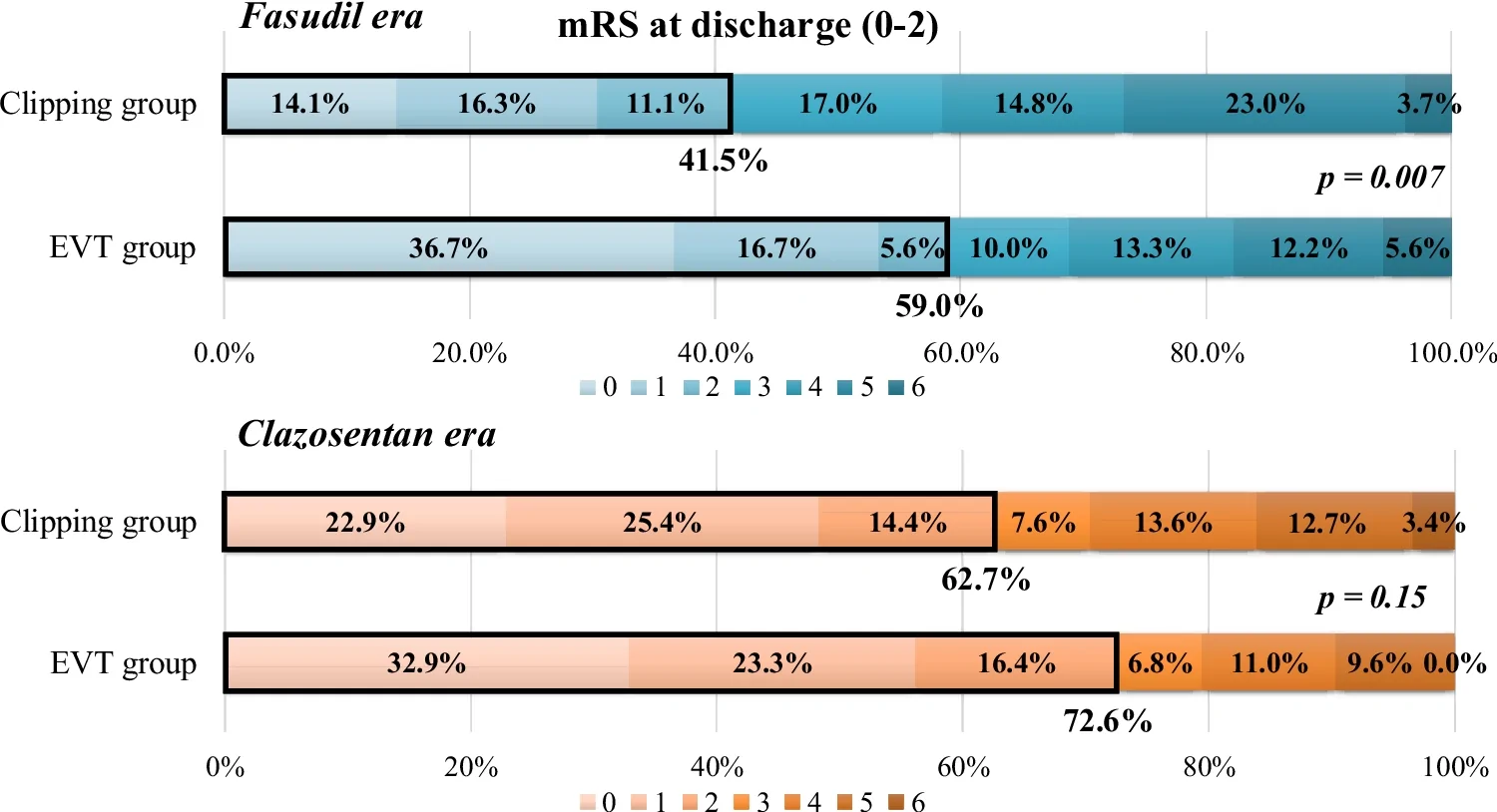
Distribution of modified Rankin Scale (mRS) scores at discharge in the fasudil and clazosentan eras. In the fasudil era, the proportion of patients with favorable outcomes (mRS 0–2) was significantly higher in the EVT group than in the clipping group (59.0% vs. 41.5%, *p* = 0.007). In the clazosentan era, both groups showed more favorable outcome distributions. Although the EVT group had a higher proportion of favorable outcomes, the difference between groups was not statistically significant (72.6% vs. 62.7%, *p* = 0.15)

Figure 2 and Tables 3 and 4 present the outcomes stratified by treatment era. In the fasudil era, favorable outcomes were significantly more frequent in the EVT group than in the clipping group (59.0% vs 41.5%, *p* = 0.007). In contrast, in the clazosentan era, no significant differences were observed between EVT and clipping groups (72.6% vs. 62.7%, *p* = 0.15). Across both treatment eras, the proportion of patients with favorable outcomes was numerically higher in the EVT group.

**Table 3:** Complications and postoperative outcomes

*Fasudil Era*	Univariate			Multivariate	
	EVT group (*n* = 90)	Clipping group (*n* = 135)	*p* value	OR (95% CI)	*p* value
Post mRS score					
mRS score, 0–2	53 (59.0%)	56 (41.5%)	0.011	3.56 (1.41–9.01)	0.007
Complications					
Cerebral vasospasm	23 (25.6%)	56 (41.5%)	0.015	0.77 (0.38–1.57)	0.48
Vasospasm-related DCI	4 (4.4%)	21 (15.6%)	0.015	0.33 (0.1–1.14)	0.079
Brain edema	4 (4.4%)	19 (14.1%)	0.027	0.42 (0.1–1.69)	0.22
Pulmonary complications	10 (11.1%)	16 (11.9%)	0.86	0.97 (0.35–2.71)	0.95
De novo ICH	1 (1.1%)	5 (3.7%)	0.27	0.85 (0.16–4.68)	0.86
Hypotension	1 (1.1%)	2 (1.5%)	0.81	0.64 (0.1–4.31)	0.65

Data are presented as n (%), as appropriate. Multivariable logistic regression adjusted for age, WFNS grade, Fisher classification, CSF drainage, and aneurysm location (anterior vs posterior circulation); Firth’s correction was applied when models did not converge. Favorable outcome was defined as mRS 0–2. *DCI* delayed cerebral ischemia, *ICH* intracranial hemorrhage, *mRS* modified rankin scale, *OR* odds ratio, *CI* confidence interval

**Table 4:** Complications and postoperative outcomes

*Clazosentan Era*	Univariate			Multivariate	
	EVT group (*n* = 73)	Clipping group (*n* = 118)	*p* value	OR (95% CI)	*p* value
Post mRS score					
mRS score, 0–2	53 (72.6%)	74 (62.7%)	0.16	1.92 (0.79–4.63)	0.15
Complications					
Cerebral vasospasm	9 (12.3%)	25 (21.2%)	0.12	0.52 (0.21–1.28)	0.16
Vasospasm-related DCI	0 (0.0%)	8 (6.8%)	0.1	0.09 (0.01–1.12)	0.061
Brain edema	0 (0.0%)	17 (14.4%)	0.026	0.03 (0–0.42)	0.009
Pulmonary complications	4 (5.5%)	13 (11.1%)	0.2	0.62 (0.17–2.17)	0.45
De novo ICH	0 (0.0%)	6 (5.1%)	0.15	0.11 (0.01–1.01)	0.051
Hypotension	0 (0.0%)	5 (4.2%)	0.189	0.21 (0.02–1.81)	0.155

Data are presented as n (%), as appropriate. Multivariable logistic regression adjusted for age, WFNS grade, Fisher classification, CSF drainage, and aneurysm location (anterior vs posterior circulation); Firth’s method was applied when models did not converge. Favorable outcome was defined as mRS 0–2. *DCI* delayed cerebral ischemia, *ICH* intracranial hemorrhage, *mRS* modified rankin scale, *OR* odds ratio, *CI* confidence interval

To assess whether the association between treatment modality and outcome differed according to drug group, we performed an interaction analysis by including a treatment modality × drug group interaction term, with drug group defined as clazosentan versus fasudil, in the multivariable model. The interaction term was not statistically significant (p for interactio*n* = 0.163). Stratified estimates derived from the interaction model showed an odds ratio of 3.58 (95% CI, 1.47–8.74) in the fasudil group and 1.42 (95% CI, 0.56–3.56) in the clazosentan group.

Thus, the apparent attenuation of the difference in favorable outcomes between EVT and clipping in the clazosentan era should be interpreted as exploratory rather than evidence of a statistically confirmed treatment-by-era effect.

### Safety outcomes

**Fig. 3:**
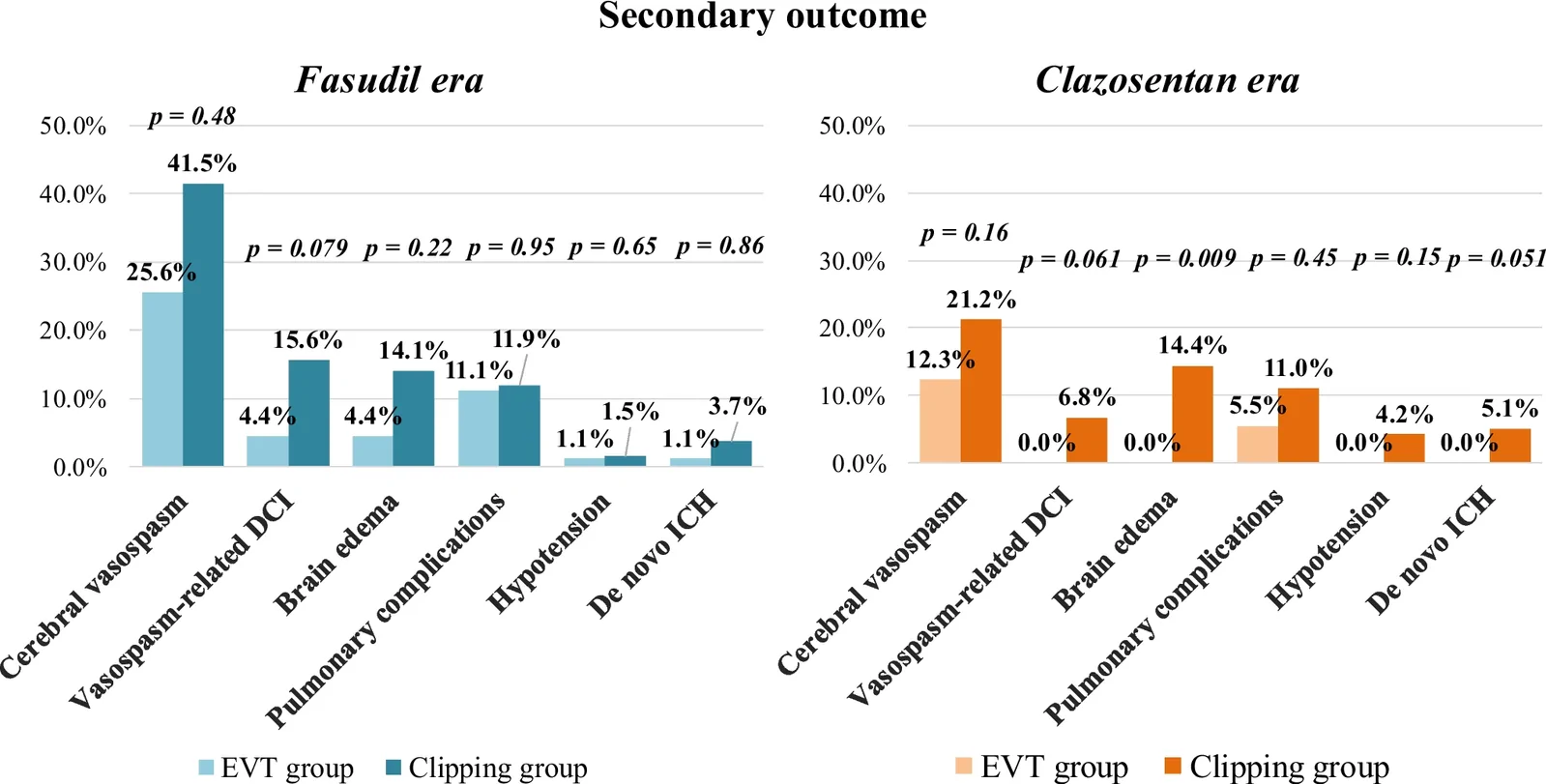
Secondary outcomes in the fasudil and clazosentan eras. The incidences of vasospasm and DCI were numerically lower in the clazosentan era than in the fasudil era. Brain edema remained significantly more frequent in the clipping group, whereas other complications showed no significant group differences

Table 2 and Fig. 3 present the analyses of secondary safety outcomes for the entire cohort. The incidences of vasospasm-related DCI, brain edema, and de novo ICH were lower in the EVT group than in the clipping group, although several of these differences did not reach statistical significance. Era-stratified analyses are shown in Tables 3 and 4 and Fig. 3.

In the fasudil era, the incidence of cerebral vasospasm was 25.6% in the EVT group and 41.5% in the clipping group, with no statistically significant difference (*p* = 0.48). The incidence of vasospasm-related DCI was 4.4% in the EVT group and 15.6% in the clipping group; although this difference was not statistically significant, the incidence was numerically lower in the EVT group (*p* = 0.079). Other complications, including brain edema, pulmonary complications, de novo ICH, and hypotension, showed no clear differences between the two groups.

In the clazosentan era, cerebral vasospasm occurred in 12.3% of the EVT group and 21.2% of the clipping group (*p* = 0.16), and DCI occurred in 0.0% and 6.8%, respectively (*p* = 0.061), with a numerically lower incidence in the EVT group but without statistical significance. Brain edema was more frequently observed in the clipping group (14.4% vs. 0.0%, *p* = 0.009). Pulmonary complications, de novo ICH, and hypotension were also more common in the clipping group, although these differences did not reach statistical significance.

Overall, era-stratified comparisons suggested lower incidences of cerebral vasospasm and vasospasm-related DCI in the clazosentan era. However, these findings should be interpreted cautiously because temporal confounding and differences in baseline characteristics between treatment eras cannot be excluded.

## Discussion

### Main findings

In our RECOVER registry data, patients treated with EVT had more favorable functional outcomes than those treated with clipping during the fasudil era. In the clazosentan era, functional outcomes appeared more favorable in both treatment groups, and the between-group difference was less pronounced in unadjusted comparisons. However, formal interaction testing using a treatment modality × drug-era term did not demonstrate statistically significant effect modification. Although stratified estimates suggested a larger adjusted treatment effect in the fasudil group than in the clazosentan group, these findings should be interpreted as exploratory given the retrospective design and limited power.

In Western countries, nimodipine has been established as an effective pharmacological agent for the prevention of cerebral vasospasm [14, 15], and meta-analyses have shown that it reduces the risks of poor outcome and delayed ischemic neurological deficits [16]. In contrast, in Japan, fasudil has been widely used as an alternative to nimodipine. Fasudil is a Rho-kinase inhibitor that has been reported to reduce the incidence and severity of vasospasm by suppressing vascular smooth muscle contraction [17, 18].

In the present study, during the fasudil era, the proportion of favorable clinical outcomes was higher in the EVT group. This finding is broadly consistent with previous randomized trials and meta-analyses that have demonstrated favorable short-term outcomes after EVT compared with clipping in selected patients with aneurysmal subarachnoid hemorrhage [2–4].

In the clazosentan era, the observed proportion of patients with favorable functional outcomes was higher in both groups than in the fasudil era.

Clazosentan has been reported not only to suppress cerebral vasospasm but also to ameliorate disturbances in distal and microvascular circulation [19]. In addition, it has been shown to be effective in preventing both proximal and distal cerebral vasospasm [20]. These pharmacological effects may contribute to a reduction in DCI. However, in the present era-based comparison, the independent contribution of clazosentan to functional outcome cannot be separated from other temporal changes in clinical practice, perioperative management, and patient selection [9, 10, 21].

Regarding DCI, the EVT group had a significantly lower incidence during the fasudil era, and although there was no statistically significant difference in the clazosentan era, the incidence was numerically lower in the EVT group. An analysis of ISAT suggested that DCI may occur slightly more frequently after clipping than after EVT [22]. Several mechanisms may account for this difference. Surgical manipulation, including brain retraction and direct handling of the subarachnoid clot and vessels, may increase the risk of vasospasm or DCI. Conversely, clipping may allow more direct evacuation of subarachnoid hematoma, which could theoretically reduce the vasospastic burden. These competing mechanisms suggest that treatment-related differences in DCI may have contributed, at least in part, to the observed outcome differences; however, the underlying mechanism remains uncertain and should be interpreted cautiously. The present findings also need to be interpreted in the context of previous international trials of clazosentan, including the CONSCIOUS series [23–26]. In those trials, higher doses of clazosentan were evaluated, and the clinical benefit on functional outcomes was limited despite reductions in angiographic vasospasm. In contrast, in real-world practice in Japan, clazosentan has generally been administered at a relatively low dose of 10 mg/h, which may reduce the risk of dose-related adverse events while preserving vasospasm-suppressive effects [9, 10, 20, 27]. However, direct comparison between the present study and previous randomized trials is difficult because of differences in study design, clazosentan dose, patient selection, background management, and outcome definitions.

### Clinical implications

Previous large randomized trials have shown that EVT is associated with more favorable short-term outcomes than clipping in selected patients with aneurysmal subarachnoid hemorrhage, although long-term concerns, including retreatment and rebleeding, have also been reported [2–8]. In the present study, functional outcomes appeared more favorable in both treatment groups during the clazosentan era. This observation may reflect advances in pharmacological therapy, but it may also be attributable to broader temporal changes in devices, operator experience, perioperative care, and institutional practice. Although participating centers were asked to follow standardized protocols, some degree of variability in imaging evaluation, vasospasm diagnosis, and postoperative management is inevitable in real-world practice. Therefore, caution is required when interpreting these results.

Moreover, the present findings are consistent with the currently widely adopted strategy of selecting EVT as the first-line treatment option when anatomically and clinically feasible. The observation that the EVT group showed higher rates of favorable functional outcomes across both eras is also in line with prior evidence [2–4, 9].

Nevertheless, clipping remains an important treatment option in selected cases. Surgical clipping may be appropriate for aneurysms with a wide neck, aneurysms that are technically difficult to treat by EVT, and cases accompanied by an intracranial hematoma requiring surgical evacuation.

The more favorable outcomes observed during the clazosentan era raise the possibility that advances in perioperative management, including pharmacological therapy, may help mitigate perioperative risks in patients who require clipping. However, pharmacological therapy should not be regarded as a primary determinant of aneurysm treatment selection, and treatment selection should continue to be based on comprehensive decision-making that incorporates aneurysm morphology, patient condition, hemorrhage characteristics, and institutional expertise. Therefore, clazosentan should be positioned not as a factor that changes the fundamental treatment strategy, but as a pharmacological adjunct that may complement perioperative management.

### Limitations

This study has several limitations. First, the comparative analysis of microsurgical clipping and EVT across pharmacological eras should be interpreted with considerable caution. Because treatment allocation was not randomized but was determined by clinical judgment in this retrospective multicenter study, substantial baseline imbalances were present between the treatment groups, limiting their comparability. Although multivariable adjustment was performed, residual confounding likely remained. Therefore, the observed differences, or lack thereof, between treatment modalities across eras may reflect selection bias rather than true differences in treatment effect or pharmacological era. In addition, potentially important factors, including detailed aneurysm morphology, hematoma volume, and institution-specific treatment strategies, were not uniformly available in the registry and therefore could not be fully adjusted for. Accordingly, these findings should be regarded as exploratory and should not be interpreted as causal.

Second, the comparison between the fasudil and clazosentan eras is inherently susceptible to temporal confounding. During the study period, advances in device technology, accumulation of operator experience, refinements in perioperative management, and changes in institutional treatment strategies may have influenced clinical outcomes. These factors may have contributed to the observed differences between eras independently of the introduction of clazosentan. Thus, the era-related findings should be interpreted as exploratory and not as evidence of the isolated effect of clazosentan.

Third, the assessment of cerebral vasospasm and delayed cerebral ischemia was based on routine clinical practice at each participating institution and was not standardized across centers. Different imaging modalities, including CTA, MRA, and DSA, were used, and variations in imaging timing and diagnostic thresholds may have introduced detection bias. Quantitative measures of interrater reliability were also unavailable. Moreover, detailed information on the frequency and timing of imaging examinations was not uniformly captured across centers; therefore, we could not formally compare imaging frequency between the clipping and EVT groups or between pharmacological eras, nor could we adjust for potential differences in surveillance intensity. Consequently, heterogeneity in imaging practices across treatment groups and study eras may have influenced the reported incidence of vasospasm and DCI.

Fourth, although clazosentan administration, including initiation, duration, discontinuation criteria, and concomitant medications, was implemented under a shared general policy, detailed practice patterns may have differed across institutions in real-world settings. Pharmacological exposure in the clazosentan era was also not uniform. Of the 191 patients treated during this era, 33 (17.3%) received concomitant fasudil, whereas 158 (82.7%) did not. Although this treatment pattern reflects real-world clinical practice, it makes it difficult to isolate the specific pharmacological effect of clazosentan and raises the possibility of confounding by indication. Because the reasons for selecting combination therapy, such as institutional protocols, disease severity, and clinician preference, were not uniformly captured in this retrospective multicenter registry, we could not perform a robust adjusted comparison between clazosentan monotherapy and combination therapy, nor could we disentangle the independent effects of each regimen.

Fifth, functional outcome was assessed only at hospital discharge. Although discharge mRS is a pragmatic endpoint in retrospective multicenter registry studies, it may be influenced by institutional differences in length of hospital stay, rehabilitation practices, and discharge policies. In addition, the absence of longer-term functional outcome data, such as 3- or 6-month mRS, limits direct comparison with prior studies such as ISAT and BRAT.

Accordingly, the present findings should be interpreted as exploratory. Further multicenter prospective studies with standardized imaging assessments, pharmacological protocols, and longitudinal follow-up are warranted to confirm the reproducibility and generalizability of our results.

## Conclusions

Functional outcomes appeared more favorable in both treatment groups during the clazosentan era. Across both pharmacological eras, the proportion of patients with favorable functional outcomes was higher in the EVT group than in the clipping group; however, formal interaction testing did not demonstrate statistically significant effect modification between treatment modality and pharmacological era. Therefore, any apparent attenuation of the outcome difference between EVT and clipping after the introduction of clazosentan should be regarded as exploratory, and no causal inference can be drawn from this retrospective era-based comparison.

These findings may reflect broader temporal changes in perioperative management, treatment strategies, and real-world practice patterns rather than the isolated effect of clazosentan. Further prospective studies with standardized treatment protocols, imaging assessments, pharmacological exposure, and longitudinal follow-up are warranted to confirm the reproducibility and clinical relevance of these findings.

## Data Availability

The datasets generated and/or analyzed during the current study are not publicly available due to ethical and institutional restrictions related to patient privacy. Clinical data were obtained under approval from the Ethics Committee of Nagoya University Hospital (approval number: 2022–0476) with an opt-out consent process. De-identified data may be made available from the corresponding author upon reasonable request and with permission from the participating institutions.
